# Does Perceptual Simulation Explain Spatial Effects in Word Categorization?

**DOI:** 10.3389/fpsyg.2019.01102

**Published:** 2019-05-17

**Authors:** Barbara Treccani, Claudio Mulatti, Simone Sulpizio, Remo Job

**Affiliations:** ^1^Department of Psychology and Cognitive Science, University of Trento, Trento, Italy; ^2^Department of Developmental Psychology and Socialisation, University of Padova, Padua, Italy; ^3^Faculty of Psychology, Vita-Salute San Raffaele University, Milan, Italy

**Keywords:** perceptual simulation, polarity correspondence account, semantic-category MARC effect, conceptual spatial compatibility, representational stimulus-response correspondence, spatial vs. symbolic compatibility, embodied cognition, word recognition

## Abstract

In three experiments we investigated the origin of the effects of the compatibility between the typical location of entities denoted by written words (e.g., “up” for *eagle* and “down” for *carpet*) and either the actual position of the words on the screen (e.g., upper vs. lower part of the screen), or the response position (e.g., upper- vs. lower- key presses) in binary categorization tasks. Contrary to predictions of the perceptual simulation account (Barsalou, 1999), conceptual spatial compatibility effects observed in the present study (faster RTs when the typical position of the stimulus referent in the real word was compatible with either the stimulus or response physical position) seem to be independent of whether there was an overlap between simulated processes possibly triggered by the presented stimulus and sensory-motor processes actually required by the task. Rather, they appear to depend critically on whether the involved stimulus and/or response dimensions had binary, variable (vs. fixed) values. Notably, no stimulus–stimulus compatibility effect was observed in Experiment 3, when the stimulus physical position was presented in a blocked design (i.e., it was kept constant within each block of trials). In contrast, in all three experiments, a compatibility effect between response position and another (non-spatial) conceptual dimension of the stimulus (i.e., its semantic category) was observed (i.e., an effect analogous to the MARC [*linguistic markedness of response codes*] effect, which is usually observed in the number domain; Nuerk et al., 2004). This pattern of results is fully accounted for by the polarity principle, according to which these effects originate from the alignment of the polarities of either different stimulus dimensions or stimulus and response dimensions.

## Introduction

The embodied cognition theory (Barsalou, 1999) has proved to be a fruitful approach to the study of language processing — the impetus to provide empirical support to this approach has indeed generated a plethora of studies and given rise to a heated debate about their findings. Yet, only some of the findings of these studies are really relevant to such an approach (cf., Mahon and Caramazza, 2008; Caramazza et al., 2014). In our view, the discrimination between relevant and misleading evidence on this issue has important theoretical consequences for explanatory hypotheses of both embodied cognition and language (and their intersection).

In this paper, we focus on the effects of objects’ typical spatial position on word recognition. We will present empirical data suggesting that conceptual spatial compatibility effects in word categorization tasks – i.e., one of the most renowned phenomena reported as evidence in support of the embodied theory of language processing – are only incidentally consistent with the embodied approach. Indeed, these phenomena appear to be more properly accounted for by an alternative view, which traces them back to more general, task-related, mechanisms of symbolic compatibility.

In an exemplar experiment showing this kind of phenomenon (e.g., Šetić and Domijan, 2007, Experiment 2) participants perform a binary discrimination task (e.g., a living/non-living classification) to words referring to entities with typical locations in the upper part of the visual field (e.g., *butterfly*) or in the lower part of the visual field (e.g., *carpet*). The actual stimulus can appear either above or below the center of the screen, thus creating congruent conditions for items with “top” typical location presented above the center of the screen and for items with “bottom” typical location presented below the center of the screen, and incongruent conditions when the pairings are reversed. What is typically found is that responses in congruent trials are faster than responses in incongruent trials.

According to several researchers, such interactions between word meaning and spatial location provide important insights into the underlying mental representations of meaning. Indeed, the idea underlying this kind of experiment is that the relationship between the spatial features coded in the word meaning, and the actual position of the word stimulus in a visual display, modulates the recognition of written words. This idea stems from accounts of embodied cognition, according to which linguistics, perceptual, and motor aspects of a word meaning are intimately related. These accounts postulate that recognizing a word requires the re-enactment of the perceptual and motor-related processing performed during actual experiences with the entity denoted by the word (e.g., Zwaan and Yaxley, 2003; Šetić and Domijan, 2007; Estes et al., 2008; Pecher et al., 2010). That is, the processing of a word involves running a simulation of an interaction with the entity to which the word refers (Barsalou, 1999; see also, e.g., Job et al., 2011; Mulatti et al., 2014, for a discussion of this hypothesis applied to the recognition of action words and pictures, respectively).

Šetić and Domijan (2007) invoked this perceptual simulation hypothesis to account for their results: participants’ mental simulations would have directed their spatial attention toward the typical location of the entity denoted by the word (i.e., upward in the case of entities with a top typical location and downward in the case of entities with a bottom typical location). This would have facilitated the processing of words when their physical position corresponded to the position toward which attention was directed.

More recently, Thornton et al. (2013) used a similar procedure to investigate whether the typical location of objects denoted by word stimuli can also affect performance when stimuli are presented at the center of the screen but response keys are vertically aligned. In this study, the authors investigated the possible effects of the compatibility between a stimulus feature (i.e., the typical position of the entity to which the word refers) and a response feature (i.e., the response position), rather than between two stimulus features (the stimulus conceptual and perceptual spatial dimensions, i.e., the typical position of the word referent and the physical position of the word). In Thornton et al.’s study, therefore, stimulus–response (S–R) correspondence, instead of stimulus–stimulus (S–S) congruency, was investigated (cf., Treccani et al., 2009).

**FIGURE 1:**
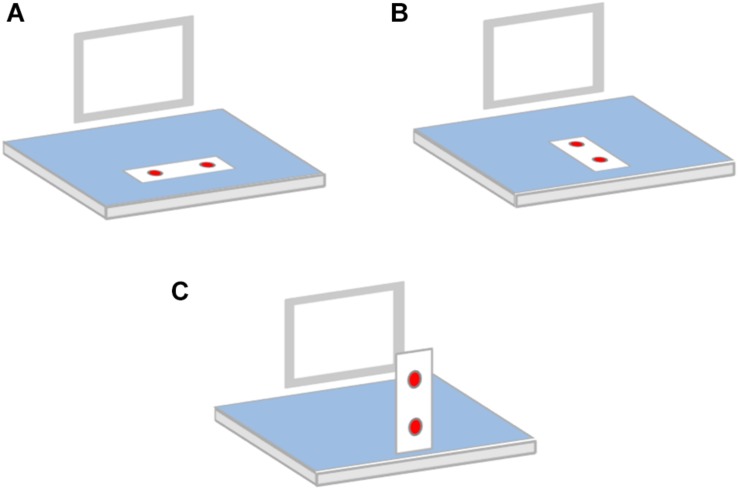
Schematic representation of response-key arrangements in our Experiments 1 and 3 **(A)**, Experiment 2 **(B)** and in Thornton et al.’s (2013) Experiment 1 **(C)**.

In their Experiment 1, Thornton et al. (2013) asked participants to categorize objects denoted by word stimuli as man-made or natural by pressing one of two keys. These keys were attached to a stand perpendicular to the table on which the computer monitor displaying the stimuli was placed (i.e., one key was above the other; see Figure 1 – panel C). Results showed that the responses were faster when objects typically associated with the upper and lower part of the visual field were responded to with the upper and lower keys, respectively, than when the opposite S–R pairings were presented (i.e., a correspondence effect of about 10 ms was observed).

S–R correspondence effects involving an interaction between word meaning and response position have been also found with horizontally aligned response keys. In this case, the conceptual size of the stimulus (i.e., the typical size of the entity to which the stimulus refers), rather than its conceptual position, has been shown to affect response speed and/or accuracy. For example, Sellaro et al. (2015) observed that, when lateralized responses are used to judge either words or pictures referring to typically large and small entities, left responses are faster for conceptually small targets, whereas right responses are faster for conceptually large targets. This kind of finding is usually explained by assuming that, even when the task does not require accessing the size of the entity to which the stimulus refers, conceptual size is spatially represented on a left-to-right oriented mental magnitude line (MML; cf., Holmes and Lourenco, 2013) in which small entities are represented on the left and large entities on the right. An S–R correspondence effect would then occur in this case between the physical position of the response and the position of the stimulus referent on the MML (cf., Treccani and Umiltà, 2011), rather than its typical position in the visual field. In this respect, these phenomena would be similar to the so-called SNARC (Spatial Numerical Association of Response Codes) effect, which is observed when target stimuli are numbers (i.e., small and large numbers are responded to faster with left and right keys, respectively; Dehaene et al., 1993): they would all be instances of a more general spatial-quantity effect (i.e., Spatial Quantity Association of Response Codes; Walsh, 2003).

All these S–R conceptual correspondence effects (both those involving numerical quantities and those involving objects’ typical position or size) can, in turn, be seen as instances of the Simon effect (Simon and Small, 1969). The Simon effect refers to the finding that responses to a non-spatial attribute of a stimulus (e.g., the color of a geometrical shape presented on the left vs. right, or on the top vs. bottom, of a computer screen) are faster and/or more accurate when its physical position corresponds to the position of the required response. From this point of view, conceptual S–R correspondence effects would be analogous to the Simon effect in a representational space, rather than in the physical space domain (Treccani et al., 2009, 2010). All these effects would result from the same basic mechanism: the correspondence between the response position and a spatial dimension of the stimulus, whether it is the stimulus’ actual physical position, the typical position of its referent in the real word, or its position on a spatially oriented medium used to represent the magnitude of the stimulus referent.

As in the case of S–S conceptual congruency effects, both S–R conceptual correspondence effects involving objects’ typical position and those involving typical size might be accounted for by perceptual simulation: following this account, the representation of the stimulus referent, or the representation of its position on the MML, might activate appropriate response-related (motor) processes, and this would facilitate responses that somehow involve the same motor processes. However, such an account raises the issue of the relevance and/or specificity of the motor responses associated to concepts (and their affordance). The referents of the stimuli used in the experiments described so far require a specific and consistent set of motor programs that differ from those required by other objects, even if they share the same typical position or the same typical size. These motor processes are, in turn, different from those involved in the responses (i.e., key presses) that they are supposed to facilitate in typical S–R conceptual correspondence paradigms. For example, grasping a chestnut involves a set of movements that are different from those involved in hanging a picture to the wall or using a lipstick. Moreover, all these different actions do not seem to have anything in common with pressing the left or upper key of a response device.

So, if perceptual simulations are at work in this kind of paradigms, what type of simulations are they? Most would agree that, if they encompass a very large and undifferentiated set of responses, say, a generic arousal to move upward (when facing stimuli with a top typical location) or leftward (when facing typically small stimuli), then the notion is too vague and too far-reaching to have explanatory value. Furthermore, there is evidence that the real-word (limb-specific) actions afforded by a target object do not affect the responses required by the task when these responses involve actions that are different from the afforded ones (e.g., when the stimuli afford reach-and-grasp actions and the task requires keypress responses): in order to have a direct effect on the required responses, the actions afforded by the stimulus must overlap with the actions involved by these responses (cf., Bub et al., 2018; see also Proctor and Miles, 2014).

Another option is to assume that sensory-motor processes triggered by stimuli in conceptual compatibility tasks (either S–S congruency or S–R correspondence tasks) do not directly affect the stimulus or response processing. According to a weaker version of the perceptual simulation account (cf., e.g., Mahon and Caramazza, 2008; Mahon, 2015), conceptual spatial compatibility effects derive from the overlap, or lack of an overlap, between spatial codes activated by the simulated sensory-motor processes and those assigned either to other aspects of the stimuli (in S–S congruency tasks) or to the required response (in S–R correspondence tasks; see Gevers et al., 2006; Santens and Gevers, 2008, for a similar, “indirect,” interpretation of the SNARC effect).

Needless to say, these codes must be abstract enough (e.g., “up” or “left”) to allow such an overlap (i.e., in order for the spatial code derived from the simulation of, e.g., watching a bird in the sky, to overlap with the way in which upper-key presses are spatially coded). Consequently, given that the conceptual compatibility effects would derive from the overlap between somehow abstract codes, one may wonder why perceptual simulation would be necessary at all to account for them.

Indeed, according to alternative accounts of conceptual compatibility effects, the stimulus and/or response codes underlying such effects do not originate from the simulation of perceptual and motor processes. Rather, they are driven by task-related factors and, actually, are not even spatial. These effects would result from the overlap between *verbal* labels assigned to stimulus and/or response values by virtue of the task structure. During the execution of the task, participants may detect symmetries and regularities in either stimulus or response values and dimensions; for example they may notice that the values of stimulus and response dimensions can all be classified in two dichotomous categories, with stimuli referring to entities which may be seen as having either “top” or “bottom” typical position, and responses referring to the pressure of either the upper or lower of two keys. That may make participants apply similar verbal labels, such as, e.g., “up” and “down,” to all the task’s relevant, and possibly irrelevant, stimulus and response values. Faster and/or more accurate responses would be observed in the case of overlapping verbal representations (cf., e.g., Gevers et al., 2010).

The overlap may occur even when the dichotomous stimulus and/or response dimensions bear no evident similarities (e.g., stimuli refer to small and large entities and responses are left- and right-key presses). According to the polarity principle (Proctor and Cho, 2006; Proctor and Xiong, 2015), stimulus dimensions with binary values are coded as having a positive or negative polarity. A clear example is provided by adjectives, for which an unmarked and a marked pole may be identified, that is, a positive, dominant, pole, referring to both the entire extension of the stimulus dimension denoted by the adjective and one of its end points (e.g., the adjectives *big* or *tall*), and a negative pole referring only to an end point of the extension (e.g., the adjectives *small* or *short*). Analogously, binary responses are encoded in terms of positive and negative polarities (e.g., when two horizontally arranged response keys are used, right and left responses would be coded as the unmarked and marked poles, respectively, of the response dimension). In forced-response reaction times, responses are faster when stimulus and response polarities, or polarities of different stimulus dimensions, are aligned (i.e., in compatible conditions) than when they are not aligned (i.e., in incompatible conditions; see also Lakens, 2011, for a discussion of the polarity vs. perceptual simulation accounts of S–S congruency effects in word recognition).

It is worth noticing that, even in the case of the Simon effect (S–R spatial correspondence effect in the physical, rather than conceptual, space domain), “direct” accounts have initially been proposed. Basically, these hypotheses maintained that spatial attention is directed toward the side of space where the stimulus appears and, because of this, the system is biased toward emitting the spatially corresponding response. For example, Simon and Small (1969) proposed that there is a basic natural tendency to respond toward the source of the stimulus: the onset of the stimulus tends to evoke a response in the direction of stimulus location. However, these hypotheses are not currently given much credence because they fail to account for the fact that an effect of the correspondence between stimulus and response locations is also observed when these locations do not rely on the viewer position (i.e., they do not refer to egocentric axes), rather, they are relative positions. This occurs, for example, when both stimuli are presented in the left hemispace, but one is on the left of the other, or when they are on the left or right with respect to a given object that acts as a reference frame (Umiltà and Liotti, 1987; Hommel and Lippa, 1995; see also the discussion about the vertical Simon effect below).

Such direct accounts have been replaced with indirect (spatial coding) accounts positing that the (relevant) response and (irrelevant) stimulus locations are spatially coded with respect to one (or more) reference frames, and response selection is faster when the response and stimulus codes correspond (see Stoffer and Umiltà, 1997). According to one of the most influential versions of the spatial coding account, the stimulus spatial code is generated because of the shift of attention toward the location occupied by the stimulus (i.e., the attention-shift account; e.g., Treccani et al., 2006) — thus attention would still be involved in the Simon effect, but its action would be indirect: the attention shift toward the stimulus is the source of the stimulus spatial code that causes the effect. However, it has been recently proposed that, at least in some circumstances, the Simon effect itself may *not* be spatial in nature and may arise from a correspondence between abstract, verbal and/or bipolar codes of stimuli and responses: even this effect might simply result from a structural correspondence between stimulus and response dimensions (cf., Proctor and Xiong, 2015).

### The Present Study

According to the stronger version of the perceptual simulation account (e.g., Šetić and Domijan, 2007), the congruency between two aspects of the stimulus (e.g., the typical position of the stimulus referent in the visual field and the physical, actual, position of the stimulus on the screen) affects the recognition of the target stimulus, whereas the correspondence between stimulus and response dimensions has an effect on the required response: the simulated processes triggered by the presented stimulus (e.g., upward movements of eyes, attention and/or limbs) would be directly involved in the analysis of the stimulus in the former case and in the to-be-emitted response in the latter case.

In contrast, according to the accounts that trace the conceptual compatibility effects back to the overlap between stimulus and/or response codes, both S–S congruency and S–R correspondence effects arise from response-selection phenomena. Irrespective of the origin of these codes (i.e., perceptual simulation or task-related mechanisms), the relationship between the codes used to represent two aspects of the target stimulus does not affect its recognition. Rather, this relationship affects the specific response-selection operations involved by the task, such as the assignment of the stimulus to task-relevant categories and the choice of the appropriate response.

Crucially, however, the hypotheses that provide for overlapping S–S or S–R codes as the underlying mechanism of conceptual compatibility effects differ with respect to the conditions that are supposed to induce stimulus and response coding. According to verbal and polarity compatibility accounts (e.g., Proctor and Cho, 2006), such coding is triggered by the structure of the task, that is, it is induced by the fact that, in tasks in which these effects are observed (i.e., binary classification tasks), stimulus and response dimensions have dichotomous values (either because they can only assume two possible values or because their values can be classified in two categories). In contrast, according to perceptual simulation accounts, this coding automatically occurs when a stimulus is presented, irrespective of the context in which it is presented.

Based on these premises, we designed three experiments that aimed to contrast the predictions of these alternative accounts. In all three experiments we used the same material (i.e., words referring to either animals or non-living things that are typically located in either the upper or lower part of the visual field) and participants had to perform the same task (i.e., a semantic decision task with bimanual responses). However, across experiments, we varied the arrangement of the response keys, the position of the target stimulus on the screen, and the context in which stimuli appeared, that is, the composition of the blocks of trials. These manipulations firstly allowed us to replicate, with the same (Italian) stimulus words, the S–S and S–R compatibility effects involving the typical position of the stimulus referent previously observed with Croatian (Šetić and Domijan, 2007) and English (Thornton et al., 2013) words (Experiments 1 and 2), and to test the idea that such effects do not necessarily result from sensory-motor simulations directly modulating either stimulus or response processing (i.e., the direct version of the perceptual simulation account), but, rather, can originate from S–S or S–R overlaps occurring at a representational level, that is, between stimulus — or stimulus and response — codes (Experiment 2). Once that had been established, these manipulations would have also allowed us to investigate whether the nature of the coding processes underlying these effects is spatial, as suggested by the indirect version of the perceptual simulation account, or symbolic/abstract, as proposed by verbal and polarity compatibility accounts (Experiment 3).

The first experiment was similar to that of Šetić and Domijan (2007). It allowed us to replicate Šetić and Domijan results in a different language and with a new set of items. Indeed, even if the congruency effect between word meaning and stimulus position observed by these authors has often been cited as one of the most prominent examples of conceptual spatial effects in word categorization tasks, there were reasons to question its robustness (cf., Thornton et al., 2013): in a subsequent study, Pecher et al. (2010) did actually fail to replicate it when using the same procedure as the original study (see also Hutchinson and Louwerse, 2013, for the finding of this effect with Hungarian material only when target stimuli referred to living entities, and Petrova et al., 2018, for the failure to replicate another conceptual spatial compatibility effect, i.e., the location-cue congruency effect, with Italian material).

The second experiment aimed instead at replicating Thornton et al.’s (2013) findings by using the same procedure and materials as in Experiment 1, except for the fact that stimuli were projected centrally on the monitor screen, and a different arrangement of response keys was used. In Experiment 1, the response device (i.e., the keyboard) was aligned horizontally (i.e., the standard arrangement was used). In Experiment 2 the keyboard was instead rotated 90°, so that the two response keys were aligned vertically. However, in this experiment, as in Experiment 1, the keyboard laid flat on the table holding the computer monitor. Therefore, unlike in Thornton et al. (2013), the two keys did not stand upright on the table but were placed on it (see Figure 1). They might be described as one above the other (i.e., one “top” key and one “bottom” key), but only because the transversal plane on which they were placed could be seen as a representation of the frontoparallel plane. One key was actually closer to the participant and the other was further away. This is indeed the typical response-key arrangement used to investigate the vertical Simon effect with stimuli presented on the top vs. bottom of the screen. The occurrence of such a S–R correspondence effect in the physical space domain suggests that people tend to code response locations on the transverse plane as top and bottom (Zhong et al., 2018), and that a correspondence effect may arise even when there is no overlap between the specific, actual, sensory-motor processes involved in stimulus analysis and response execution, but there is an overlap between the way in which stimuli and responses are represented (see previous discussion about direct and indirect accounts of the Simon effect).

Accordingly, the finding of a S–R correspondence effect in our Experiment 2 (i.e., faster RTs when words referring to typically “up” and “down” entities were responded to with the top and bottom keys, respectively) could not be accounted for by the “direct” (strong) version of the perceptual simulation account (Barsalou, 1999; Šetić and Domijan, 2007): the motor simulations that might be triggered by the processing of word stimuli (e.g., movements toward the upper part of the visual field) could in no way be involved in the processes required to emit the actual response (e.g., pressing the further of the two response keys). S–R correspondence can only occur at a representational level, and an overlap between stimulus and response codes must necessarily be invoked to account for the possible S–R correspondence effect.

In Experiment 3, stimuli were again presented at the top or at the bottom of the screen. Unlike in Experiment 1, however, we used a blocked design for the presentation of stimuli, so that in a block of trials all stimuli were presented, for instance, at the top of the screen and in a second block they were all presented at the bottom of the screen. This latter experiment was crucial as, in it, both the strong and weak versions of the account based on perceptual simulation (Barsalou, 1999; Šetić and Domijan, 2007) make different predictions from those made by verbal and polarity compatibility accounts.

According to the perceptual simulation account, “up” and “down” meanings are necessarily activated by the processes required for the recognition of word stimuli referring to objects typically located in either the upper or lower part of the visual field, irrespective of the composition of the block of trials in which such stimuli are presented. The processing of, say, the word AEROPLANE requires the re-enactment of perceptual and motor aspects of previous experiences with the same kind of object (experiences related to actions like glancing up, pointing to the sky, etc.), which would facilitate upward sensory-motor processes, such as the movement of attention and/or glance from the center of the screen toward the word stimulus when it appears on the top of the screen, and possibly interfere with downward processes. It might also be the other way round: the movement of attention/eye gaze toward a stimulus on the top of the screen would activate the same processes (and the same cerebral areas) as those involved in previous experiences with examples of the target category (i.e., airplanes), which lead to a more efficient processing of the word AIRPLANE compared to words referring to objects typically located in the lower part of the visual field (e.g., the word SNAKE). Alternatively, one may think that the sensory-motor simulations triggered by the processing of the word AIRPLANE lead to the activation of a *spatial* code (i.e., “Up”) which may be either congruent or incongruent with that activated by the actual movement of attention/glance toward the word. Recognition of the word would be facilitated in the case of congruency between the two codes. Both direct and indirect versions of the perceptual simulation account, therefore, predict that S–S congruency effects are observed irrespective of the structure of the task in which stimuli appear and even if the location of the stimuli is presented in a blocked design.

Conversely, verbal and polarity compatibility accounts predict no congruency effects in Experiment 3, as the layout of the stimulus display (i.e., blocked stimulus location) prevented coding of location in terms of opposite verbal or bipolar codes (i.e., “up” vs. down” or “+polar” vs. “−polar” codes). Given that the stimulus location on the screen had only one fixed value in the block of trials in which stimuli appeared, no binary (e.g., bipolar) coding of this critical stimulus dimensions could occur, and no overlaps between different stimulus codes could take place that might cause S–S congruency effects (see Ansorge and Wühr, 2004, for evidence of no Simon effect in a RT discrimination task involving stimuli with binary – task relevant and irrelevant –attributes when the response dimension, instead of the stimulus dimension, has only one value; i.e., in a go/no go task; see also Sellaro et al., 2013).

## Materials and Methods

### Participants

Twenty-four (10 males, mean age: 21.09), 12 (4 males, mean age: 20.92), and 24 (8 males, mean age: 27.75) university students took part in Experiments 1, 2, and 3, respectively^1^. Participation was on voluntary basis. All participants were native Italian speakers with normal or corrected-to-normal vision.

### Materials and Procedure

A set of 80 words was selected (see Supplementary Material). Half of them were names of animals and half were names of non-living things. For each semantic category, half of the words had referents associated with an “up” position (e.g., HAWK and ROOF), and half with a “down” position (e.g., MOLE and CARPET). Target words were presented in black (Courier new 18-point bold font) on a white background.

Participants were tested individually. Their task was to indicate whether each of the presented words was the name of a living or non-living entity.

Stimulus presentation was controlled by E-Prime Software. A fixation point (+++) appeared in the center of the screen. In Experiment 2, it was visible for 500 ms, and was followed by a blank interval of 100 ms. In Experiments 1 and 3, as in the Šetić and Domijan (2007) study, it was visible for 300 ms and then was moved toward either the top or the bottom of the screen in two steps (both of 300 ms). In all the experiments, the stimulus appeared in the position cued by the fixation point (i.e., at the center of the screen in Experiment 2, and either 6° above or below the center of the screen in Experiments 1 and 3) and remained visible until response, or for a maximum of 3,000 ms (Experiments 1 and 3) or 1,200 ms (Experiment 2); a shorter response deadline was sufficient for Experiment 2 as no attentional shifts toward the stimulus were required. The inter-trial interval was 800 ms. Visual feedback was provided in the case of an error.

Responses were given by pressing one of two keys (the letters “m” and “z” of the keyboard) that were operated with the index fingers of the two hands. In Experiments 1 and 3, the keyboard was horizontally aligned, thus the “m” and “z” keys were operated with the left and right index fingers, respectively. In Experiment 2, the keyboard was turned 90° clockwise with respect to the standard arrangement, so that the “m” key was on the top of the keyboard (i.e., further from the participant) and the “z” key” was on the bottom of the keyboard (i.e., closer to the participant). Half of the participants operated the “m” and “z” keys with the left and right index fingers, respectively, while the opposite assignment was given to the other half. In all the experiments, the mapping of semantic category (i.e., living vs. non-living) to response keys (“m” and “z”) was counterbalanced across participants: half of the participants responded to living and non-living entities with the “m” and “z” keys, respectively, while the opposite mapping was given to the other half.

In all the experiments, the 80 stimulus words appeared twice, which resulted in a total number of 160 trials. In Experiments 1 and 3, each stimulus appeared once on the top and once on the bottom of the screen. In Experiment 1, words appeared randomly either on the top or the bottom. In Experiment 3, where a blocked design was used, all words appeared in the top position in a block of trials and in the bottom position in another block. In this experiment, the order of the two blocks of trials (i.e., only-top and only-bottom blocks) was counterbalanced across participants.

In all the experiments, experimental trials were preceded by 10 practice trials^2^.

### Ethics Statement

This study was carried out in accordance with the recommendations of the ethical committee of the University of Padova and with the Declaration of Helsinki. The study only involved adult participants and, in it, identifiable human data were not recorded. All subjects were given an information sheet which described the purpose of the study and the way in which collected data would have been processed, stored and presented in final reports of the study (i.e., only in aggregate forms). Oral informed consent was obtained from all participants. The experiments described here were part of a larger research project involving the recording of reaction times to word stimuli. The protocol of this project, including the consent procedure, was approved by the ethical committee of the University of Padova.

## Results

The effect on response times (RTs) of the compatibility (either S–S congruency or S–R correspondence) between the typical position of the object to which the word referred and either the position of the stimulus on the screen (Experiments 1 and 3) or the position of the response key (Experiment 2) was analyzed by means of one-way ANOVAs with Compatibility (compatible vs. incompatible) as within-subjects or within-items factor.

Errors (i.e., either omissions or presses of the key that corresponded to the alternative, incorrect, response; 4.3, 7.2, and 2.9% in Experiments 1, 2, and 3, respectively) and outliers (1.3, 1.5, and 1.6% in Experiments 1, 2, and 3, respectively) were excluded from the RT analyses. Outliers were identified with the van Selst and Jolicoeur’s (1994) procedure.

**FIGURE 2:**
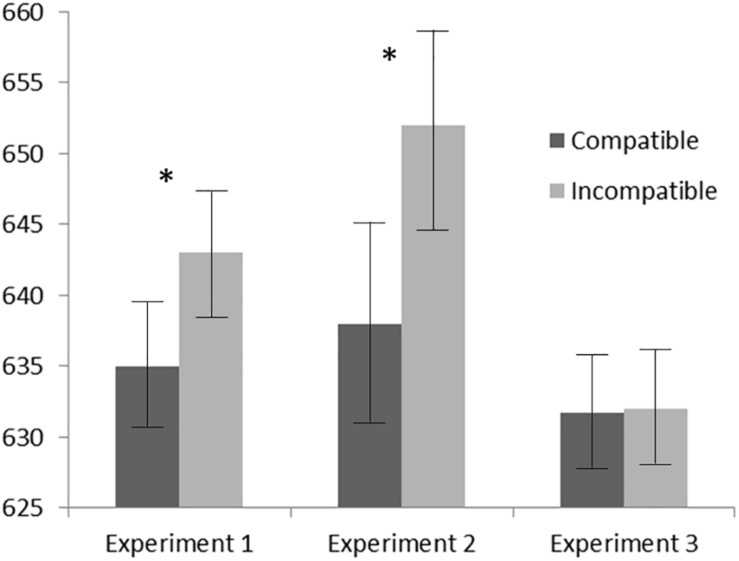
Mean correct response times (RTs) as a function of the compatibility between the typical position of the word referent and either the word stimulus position on the screen (Experiments 1 and 3) or the response position (Experiment 2). Error bars represent within-subjects 95% confidence intervals (Loftus and Masson, 1994). Asterisks indicate a significant difference (*p* < 0.05) between the two conditions (compatible and incompatible trials).

ANOVAs on RTs showed significant compatibility effects in both Experiment 1 (*F*_subjects__1,23_ = 5.49, *p* = 0.028, $\eta_{p}^{2}$ = 0.19; *F*_items__1,79_ = 4.7, *p* = 0.032, $\eta_{p}^{2}$ = 0.06) and Experiment 2 (*F*_subjects  1,11_ = 8.33, *p* = 0.015, $\eta_{p}^{2}$ = 0.43; *F*_items 1,79_ = 4.4, *p* = 0.039, $\eta_{p}^{2}$ = 0.05), but a lack of a compatibility effect in Experiment 3 (*F*_subjects__1,23_ = 0.01, *p* = 0.92, $\eta_{p}^{2}$ = 0.0004; *F*_items__1,79_ = 0.18, *p* = 0.671, $\eta_{p}^{2}$ = 0.002). As shown in Figure 2, in Experiments 1 and 2 responses were faster when the typical position of the stimulus referent was compatible with the physical position of either the stimulus (Experiment 1) or the required response (Experiment 2) than when they were incompatible (636 vs. 643 ms for Experiment 1 and 638 vs. 652 ms for Experiment 2). No difference between compatible and incompatible mean RTs (both 632 ms) was found in Experiment 3.

One-tailed comparisons between the compatibility (S–S congruency) effects of Experiments 1 and 3 (i.e., the difference between incongruent and congruent trials when stimulus location was variable and blocked, respectively) reveal that, as expected, the effect observed in Experiment 1 was significantly larger than the 0-ms effect of Experiment 3 (*t*_subjects 46_ = 1.73, *p* = 0.045; *t*_items__79_ = 1.99, *p* = 0.025).

The same ANOVAs were performed on error percentages. No significant effects were found in these analyses: Experiment 1 (*F*_subjects__1,23_ = 0.00, *p* = 1.00, $\eta_{p}^{2}$ = 0.000; *F*_items__1,79_ = 0.00, *p* = 1.00, $\eta_{p}^{2}$ = 0.000), Experiment 2 (*F*_subjects  1,11_ = 0.06, *p* = 0.819, $\eta_{p}^{2}$ = 0.005; *F*_items 1,79_ = 0.10, *p* = 0.751, $\eta_{p}^{2}$ = 0.001), Experiment 3 (*F*_subjects__1,23_ = 0.21, *p* = 0.652, $\eta_{p}^{2}$ = 0.009; *F*_items__1,79_ = 0.18, *p* = 0.676, $\eta_{p}^{2}$ = 0.002).

### Ad-Interim Discussion and Additional Analyses

As explained in the Introduction, according to the embodied accounts of spatial compatibility effects in word categorization tasks, significant S–S congruency effects should be found in *both* Experiments 1 and 3 (a significant S–R correspondence effect in Experiment 2 should also be found according to the direct, but not the indirect, version of the perceptual simulation hypothesis). Therefore, the lack of a significant S–S congruency effect in Experiment 3 was not predicted by these accounts. In contrast, the results of *all* experiments are consistent with verbal and polarity accounts of these effects. According to these accounts, significant compatibility effects should be observed in Experiments 1 and 2, whereas no effect of the congruency between stimulus location and stimulus meaning should emerge in Experiment 3, given that the location of the stimulus on the screen, being fixed within each block of trials, could not be represented in terms of binary codes (i.e., “up” vs. “down”. or “+polar” vs. “−polar”) and no overlap between this stimulus dimension and the typical position of the referent of the word could occur.

Yet, the polarity version of these accounts predicts that, in Experiment 3 too, spatial stimulus and/or response features (e.g., the left vs. right position of the response key) can interact with *other* stimulus and/or response dimensions, that, albeit not having any spatial meaning, have dichotomous values that can be coded in terms of positive and negative poles. Based on this, we decided to evaluate in Experiment 3 the occurrence of an effect that was unequivocally attributable to the polarity principle. That would have allowed us to rule out possible methodological flaws of this experiment. Indeed, the lack of a significant spatial congruency effect involving the stimulus perceptual and conceptual spatial dimensions in Experiment 3 might be traced back to the poor sensitivity of the methods used in this experiment to either collect or analyze data. The finding of another (significant) compatibility effect would have made this possibility unlikely.

Further (unplanned) analyses were then performed on data from Experiment 3 to evaluate the polarity hypothesis by testing the effect of the correspondence between the response position and a non-spatial stimulus dimension, which, in contrast with stimulus location, varied within each block of trials: the word semantic category. Indeed, the former endpoints of the (stimulus) living vs. non-living and (response) right vs. left dimensions should be both coded as the positive poles and the latter as the negative poles of these dimensions (Proctor and Cho, 2006). A polarity S–R correspondence effect might then be observed that involved these dimensions. In order to test this hypothesis, we ran a by-items analysis with Semantic Category as between-items factor and Response Key as within-item factor. The analysis showed a main effect of Response Key, *F*_1__.__7__8_ = 5.1, *p* = 0.027, $\eta_{p}^{2}$ = 0.06 a main effect of Semantic Category, *F*_1__.__7__8_ = 11.2, *p* = 0.001, $\eta_{p}^{2}$ = 0.13, and, crucially, a significant interaction, *F*_1__.__78_ = 67.8, *p* < 0.001, $\eta_{p}^{2}$ = 0.46: Responses to living entities were faster with the right than with the left hand (614 vs. 638 ms) and responses to non-living entities were faster with the left than with the right hand (637 vs. 679 ms).

These results indicate that, although the performance of participants in Experiment 3 was not affected by the congruency between perceptual and conceptual stimulus dimensions, it was nonetheless affected by the compatibility between other critical task dimensions; we observed a correspondence effect that involved the polarities of the stimulus semantic category and response position.

This effect is reminiscent of a well-known S–R correspondence effect that is usually observed in number categorization tasks, that is, the MARC (linguistic markedness of response codes) effect (Nuerk et al., 2004): when the parity status of centrally presented digits has to be judged by pressing either a left- or right- key, responses are faster when even (i.e., the unmarked, positive, pole of the number parity dimension) numbers correspond to a right keypresses and odd numbers correspond to a left keypresses, than when opposite S–R mappings are assigned to participants. This effect, just as the effect of the correspondence between the word semantic category and response position observed here, can only be explained by the polarity principle, that is, by the correspondence between the polarity, or “linguistic markedness,” of the stimulus and response codes.

In order to control for the presence of this polarity (MARC-like) correspondence effect in the other two experiments, the same analysis involving response position and semantic category done for Experiment 3 was performed for Experiments 1 and 2 (for Experiment 2, +polar and −polar responses were top and bottom keypresses, respectively). These analyses revealed the same significant effects as those observed for Experiment 3. In both experiments, we found main effects of Response position and Semantic Category, *F*_1__,__78_ = 5.0, *p* = 0.028, $\eta_{p}^{2}$ = 0.06, and *F*_1__,__78_ = 16.4, *p* < 0.001, $\eta_{p}^{2}$ = 0.17 (Experiment 1), *F*_1__,__78_ = 11.3, *p* = 0.001, $\eta_{p}^{2}$ = 0.13, and *F*_1__,__78_ = 4.1, *p* = 0.047, $\eta_{p}^{2}$ = 0.05 (Experiment 2). More importantly, the critical interaction between these two factors were observed in both Experiment 1, *F*_1__,__78_ = 230.3, *p* < 0.001, $\eta_{p}^{2}$ = 0.75, and Experiment 2, *F*_1__,__78_ = 33.8, *p* < 0.001, $\eta_{p}^{2}$ = 0.30. Either right or top keypresses were faster than left or bottom keypresses when responding to living entities (596 vs. 669 ms and 615 vs. 654 ms in Experiments 1 and 2, respectively), whereas left or bottom keypresses were faster than right or top keypresses when target stimuli referred to non-living entities (624 vs. 721 ms and 659 vs. 678 ms in Experiments 1 and 2, respectively).

## Discussion

In three experiments, we have shown that participants’ performance can be affected by the compatibility between a conceptual stimulus feature and a physical (spatial) feature of the stimulus/response arrangements.

In Experiments 1 and 2, the conceptual dimension showing the effect was the typical location of the entity to which the word referred. This conceptual spatial dimension interacted either with another (perceptual) stimulus spatial dimension (i.e., the actual location of the word stimulus referring to that concept; Experiment 1) or with a response spatial dimension (i.e., the position of the response keys; Experiment 2): faster RTs were observed when the typical location of the stimulus referent in the real word was congruent with the stimulus physical location on the screen or corresponded to the position of the required response.

These two experiments replicated the studies by Šetić and Domijan (2007) and by Thornton et al. (2013), respectively, in a different language and with a new set of items. Interestingly, the two experiments used the same materials, and it is reasonable to conclude that the observed effects result from the same type of processes, which involve two stimulus dimensions in the former case, and a stimulus dimension and a response dimension in the latter. The S–R conceptual spatial correspondence effect of Experiment 2 was indeed simply obtained by removing the stimulus perceptual spatial dimension (i.e., word stimuli only appeared at the center of the screen) and by rotating the response device so as to allow an overlap between the stimulus conceptual spatial dimension and the response position: both the typical position of the word referent and the position of the response key could be described as either “top” or “bottom”. However, in this experiment, the S–R overlap can only occur at a representational level, given that the two response keys were actually placed on the horizontal plane, rather than on the frontoparallel plane. Therefore, the S–R correspondence effect observed in Experiment 2 does not seem to be accountable for by a strong (direct) version of the perceptual simulation account. The possible motor processes evoked by the simulations of an actual interaction with the referent of the word stimulus cannot be involved in the responses required by the task (i.e., pressing either the closer or further of two keys). No upward/downward movements, or even just movements from one location to another, were required in this experiment, as well as in any other experiment of our study.

An explanation involving an overlap between stimulus and response codes seems to account better for the conceptual compatibility effects observed in the first two experiments. Results of Experiment 3 may shed light on the nature and origin of these codes.

In Experiment 3, as in Experiment 1, word stimuli with a spatial attribute (their position on the screen) were used, but the simple manipulation of blocking stimulus physical position across trials caused the effect of the other (conceptual) spatial attribute of stimuli (i.e., the typical position of the word referent) to disappear. In contrast, another conceptual stimulus dimension (i.e., the word semantic category) affected the speed with which the two alternative responses (i.e., left- and right-key presses) were performed. On the whole, in all three experiments, the aspect of the stimulus and/or response dimensions that proved to be critical in inducing compatibility effects is the fact that these dimensions have variable, binary (vs. fixed) values. Our results can hardly be accounted for by the perceptual simulation hypothesis, not even by its weaker version, that is, by assuming that processes triggered by sensory-motor simulations lead to stimulus spatial coding that may facilitate the recognition of the stimulus or the selection of the appropriate response. According to this hypothesis, indeed, such a spatial coding should be automatic in nature and occur regardless of the structure of the task in which the stimulus is presented.

In contrast, this pattern of results is nicely accounted for by binary abstract (i.e., non-spatial) coding accounts of conceptual compatibility effects in word categorization tasks. In particular, all the observed compatibility effects can be explained by the polarity principle (Proctor and Cho, 2006), according to which these effects stem from the structural similarity between stimulus and/or response dimensions. A compatibility effect would occur when an alignment between critical task dimensions is possible, because these dimensions have an asymmetric structure and both a positive and negative pole can be associated to stimulus and/or response alternatives. In Experiments 1 and 2, a compatible effect was observed that can be accounted for by the alignment between the polarities of a conceptual dimension of the target stimulus (i.e., the position implicitly meant by the word) and the polarities of either the stimulus or response actual position. In contrast, no compatibility effect would occur when the arrangement of stimuli and/or responses is such that the alignment between two task dimensions is not possible and/or feasible. As shown by Experiment 3, a compatibility effect occurs only for stimulus or response attributes providing for two alternatives within the same block of trials, that is, stimulus or response dimensions with binary values: a polarity correspondence effect was observed between the word semantic category and the response position, whereas no congruency effect was observed that involved the word stimulus actual position and the typical position of the object to which the word referred. The polarity correspondence effect observed in Experiment 3 is analogous to the MARC effect in the number domain (i.e., a correspondence effect between the polarities of the relevant stimulus and response dimensions in parity judgment tasks, i.e., response position and number parity status; Nuerk et al., 2004) and was also observed in Experiments 1 and 2. To the best of our knowledge, this is the first time that such an effect is observed in a non-numerical domain, with written words as stimuli, and in combination with other compatibility effects. As suggested above, all these compatibility effects (both those involving the stimulus conceptual position and those involving stimulus semantic category) might actually be the same effect, involving different dimensions of stimuli and responses, and can be traced back to the same (polarity) principle.

This account explains the pattern of data in a very direct manner. In addition, it collocates the phenomenon under discussion in the broader class of symbolic S–S and S–R compatibility effects (Simon et al., 1981; Kornblum, 1992), allowing an account in terms of very basic and pervasive mechanisms (cf., Proctor and Xiong, 2015).

Some caution should be taken in drawing such conclusions, though. In Experiment 3, we did not find a significant effect of the congruency between stimulus physical and conceptual positions (either in the by-subject or by-item analysis). Indeed, we found exactly the same mean RTs in congruent and incongruent conditions (i.e., a completely zero effect was observed). However, this null effect was observed in a relatively underpowered experiment (see Footnote 1). The effect of Experiment 1 proved to be significantly larger than that in Experiment 3, but this may simply mean that the polarity compatibility *contributed* to the incongruent–congruent RT difference observed in Experiment 1 (i.e., we cannot completely rule out that such a difference *also* reflects a truly *spatial* congruency effect, which is not linked to the binary structure of the task, that went undetected in Experiment 3).

Moreover, the experimental manipulation used in Experiment 1 vs. 3 (i.e., presenting the stimulus position in a mixed/variable vs. blocked design) proved to modulate the S–S conceptual congruency effects observed in these experiments, but such a manipulation was not used in the case of the S–R correspondence task of Experiment 2. It is still unknown, therefore, whether the conclusions drawn on the basis of the results of Experiment 3 (i.e., either a reduction or elimination of the effect when, in a given block of trials, stimulus physical position assumes only one possible value) can be generalize to S–R conceptual correspondence effects.

Finally, the small numbers of participants did not allow us to analyze jointly the two types of conceptual compatibility effects observed in the present study: the compatibility effects involving the conceptual stimulus position and that involving the stimulus semantic category. Actually, the experiments of this study were not specifically designed to test this latter effect. Future studies should be conducted to address this issue and explore further the relation (i.e., similarities and possible differences) between these phenomena.

## Conclusion

Results of our study call into question the causal role of perceptual simulation as either the direct or indirect determinant of the conceptual effects observed in these experiments and in previous studies using similar paradigms. The present results suggest that such effects result from ad-hoc strategies in dealing with this kind of task (i.e., word categorization tasks), which, in turn, affect operations that are specific to these tasks (i.e., processes involved in binary classifications). Therefore, they also call into question the idea that these effects are automatic in nature and that, in S–S conceptual congruency tasks (i.e., Šetić and Domijan, 2007), the congruency between the position on the visual display of the word referring to an object and the typical location of this object has an effect on word recognition processes. Even with the caveats described above, verbal and polarity hypotheses seem a more consistent account of the conceptual compatibility effects observed in the present study. According to these hypotheses, all the observed conceptual effects are attributable to (the same) response-selection phenomena.

The evidence presented here does not rule out effects of embodiment in lexical processing (see, e.g., Hauk et al., 2004). What the present results suggest, however, is that the compatibility effect between a concept typical position and either the actual location of the stimulus on the vertical dimension or the response position is not one of such embodiment effects (see also Petrova et al., 2018, for similar conclusions concerning the location-cue conceptual congruency effect originally observed by Estes et al., 2008). Further studies are needed to understand better the nature of these phenomena and to investigate whether truly embodiment effects can be observed by using variations of these paradigms (i.e., tasks that do not involve the binary classification of stimuli by means of two-choice keypress responses).

## Ethics Statement

This study was carried out in accordance with the recommendations of the ethical committee of the University of Padova. All subjects gave informed consent in accordance with the Declaration of Helsinki.

## Author Contributions

BT, CM, SS, and RJ contributed to the conception and design of the study. BT, CM, and SS collected data and performed the statistical analyses. BT drafted the manuscript. All authors reviewed, read, and approved the submitted version.

## Conflict of Interest Statement

The authors declare that the research was conducted in the absence of any commercial or financial relationships that could be construed as a potential conflict of interest.

## Footnotes

1 We planned to test 12 participants in Experiment 2. Based on the relatively large effect sizes reported for S–R conceptual correspondence effects (e.g., SNARC-like effects), this number of participants seemed to be adequate. We doubled the number of participants to test the S–S conceptual congruency effect in Experiment 3 (i.e., the experiment for which a null effect was predicted by verbal and polarity compatibility accounts) and in Experiment 1 (i.e., the experiment the results of which should be compared to those of Experiment 3).After data collection, we conducted power analyses using G*power. These analyses revealed that, with 24 participants (the number of participants in both Experiments 1 and 3), we had 0.61 probability of detecting the S–S congruency effect observed in Experiment 1. The power (1-β error probability) of Experiment 2 was instead 0.75.

2 The task used in our Experiments 1 and 3 were very similar to that of Šetić and Domijan (2007) Experiment 2. Minor differences are a slightly shorter (i.e., 500 ms instead of 800 ms) inter-trial interval (i.e., the interval between the participant’s response in a trial and the onset of the fixation point of the next trial) and the color of the stimuli, which in Šetić and Domijan Experiment 2 appeared in yellow font on a blue background. In Šetić and Domijan Experiment 2 there were no time limit for responding. However, we gave participants a lot of time to respond (up to 3,000 ms), which proved to be largely sufficient: in either Experiment 1 or 3, there were no omissions.The main difference between the S–R correspondence task of our Experiment 2 and Thornton et al.’s (2013) Experiment 1 is the arrangement of the response keys (see Figure 1). However, there were other minor differences, such as the fact that, in Thornton et al.’s (2013) experiment, the responding fingers were the thumbs and the semantic-category-to-response-key mapping was counterbalanced within participants (i.e., all participants were assigned with a mappings in a block of trials and with the opposite mapping in the other block of trials), instead of across participants. Moreover, Thornton et al. (2013) used a fixed inter-target interval (2,000 ms) and a variable inter-trial interval: after a 1,000-ms central fixation cross, the word stimulus appeared for 1,000 ms and was followed by a blank interval of other 1,000 ms. Participants’ responses did not interrupt the stimulus presentation and no feedback was presented (responses that exceed 2,000 ms, i.e., those emitted when the next trial was already started, were rejected by the experimental software and the trial was repeated).
