# Supplementary material for: Does Perceptual Simulation Explain Spatial Effects in Word Categorization?
Source: Front Psychol. 2019 May 17;10:1102. doi: 10.3389/fpsyg.2019.01102 (PMC6533881; doi:10.3389/fpsyg.2019.01102)
Supplement: Supplementary file 1 [file Table_1.DOCX]

**List of the stimuli (Italian words) used in Experiments 1-3.**

| Semantic Category | Typical Position | Stimulus (English translation) |
| --- | --- | --- |
| Animal | Up | MOSCA (FLY) |
| Animal | Up | PETTIROSSO (ROBIN) |
| Animal | Up | PICCHIO (WOODPECKER) |
| Animal | Up | FARFALLA (BUTTERFLY) |
| Animal | Up | PIPISTRELLO (BAT) |
| Animal | Up | AQUILA (EAGLE) |
| Animal | Up | CALABRONE (HORNET) |
| Animal | Up | RONDINE (SWALLOW) |
| Animal | Up | CORNACCHIA (HOODED CROW) |
| Animal | Up | GABBIANO (SEAGULL) |
| Animal | Up | CORVO (CARRION CROW) |
| Animal | Up | ALBATROSS (ALBATROSS) |
| Animal | Up | FALCO (HAWK) |
| Animal | Up | AIRONE (HERON) |
| Animal | Up | CICOGNA (STORK) |
| Animal | Up | MERLO (BLACKBIRD) |
| Animal | Up | CONDOR (CONDOR) |
| Animal | Up | AVVOLTOIO (VULTURE) |
| Animal | Up | GUFO (OWL) |
| Animal | Up | FALCONE (FALCON) |
| Animal | Down | SERPENTE (SNAKE) |
| Animal | Down | LOMBRICO (EARTHWORM) |
| Animal | Down | VERME (WORM) |
| Animal | Down | LUMACA (SNAIL) |
| Animal | Down | LUCERTOLA (LIZARD) |
| Animal | Down | BRUCO (CATERPILLAR) |
| Animal | Down | GRANCHIO (CRAB) |
| Animal | Down | SCORPIONE (SCORPIO) |
| Animal | Down | TOPO (MOUSE) |
| Animal | Down | TALPA (MOLE) |
| Animal | Down | CRICETO (HAMSTER) |
| Animal | Down | CONIGLIO (RABBIT) |
| Animal | Down | RICCIO (HEDGEHOG) |
| Animal | Down | FORMICA (ANT) |
| Animal | Down | TASSO (BADGER) |
| Animal | Down | LEPRE (HARE) |
| Animal | Down | PUZZOLA (SKUNK) |
| Animal | Down | PROCIONE (RACCOON) |
| Animal | Down | CASTORO (BEAVER) |
| Animal | Down | SALAMANDRA (SALAMANDER) |
| Non Living | Up | CIELO (SKY) |
| Non Living | Up | ARCOBALENO (RAINBOW) |
| Non Living | Up | SOLE (SUN) |
| Non Living | Up | LUNA (MOON) |
| Non Living | Up | NUVOLA (CLOUD) |
| Non Living | Up | AEROPLANO (AIRPLANE) |
| Non Living | Up | ELICOTTERO (HELICOPTER) |
| Non Living | Up | TORRETTA (TURRET) |
| Non Living | Up | LAMPADARIO (CHANDELIER) |
| Non Living | Up | CAMPANILE (BELL TOWER) |
| Non Living | Up | RAZZO (ROCKET) |
| Non Living | Up | AURORA (AURORA) |
| Non Living | Up | ATTICO (ATTIC) |
| Non Living | Up | SOFFITTO (CEILING) |
| Non Living | Up | TETTO (ROOF) |
| Non Living | Up | SATELLITE (SATELLITE) |
| Non Living | Up | METEORA (METEOR) |
| Non Living | Up | COMIGNOLO (CHIMNEY) |
| Non Living | Up | VETTA (SUMMIT) |
| Non Living | Up | ANTENNA (ANTENNA) |
| Non Living | Down | ASFALTO (ASPHALT) |
| Non Living | Down | CANALE (CHANNEL) |
| Non Living | Down | TAPPETO (CARPET) |
| Non Living | Down | PARQUET (PARQUET) |
| Non Living | Down | POZZO (WATER WELL) |
| Non Living | Down | SOTTOMARINO (SUBMARINE) |
| Non Living | Down | MINIERA (MINE) |
| Non Living | Down | BUCA (HOLE) |
| Non Living | Down | FIUME (RIVER) |
| Non Living | Down | ZERBINO (MAT) |
| Non Living | Down | STRADA (STREET) |
| Non Living | Down | PAVIMENTO (FLOOR) |
| Non Living | Down | CANTINA (CELLAR) |
| Non Living | Down | SEMINTERRATO (BASEMENT) |
| Non Living | Down | SUOLO (SOIL) |
| Non Living | Down | SCARPA (SHOE) |
| Non Living | Down | FANGO (MUD) |
| Non Living | Down | MARCIAPIEDE (SIDEWALK) |
| Non Living | Down | SENTIERO (PATHWAY) |
| Non Living | Down | BINARI (TRACKS) |
